# Study of Haloperidol for Abdominal Pain in the Emergency Department (SHAPE)

**DOI:** 10.5811/westjem.2021.2.50390

**Published:** 2021-05-05

**Authors:** Katherine Knudsen-Lachendro, Kyle Stith, Laine A. Vicarel, Brittany Harbert, Baruch S. Fertel

**Affiliations:** * Cleveland Clinic Lutheran Hospital, Department of Pharmacy, Cleveland, Ohio; † Cleveland Clinic Medina Hospital, Department of Pharmacy, Medina, Ohio; ‡ Aultman Hospital, Department of Pharmacy, Canton, Ohio; § Cleveland Clinic Enterprise Services Institute, Enterprise Quality and Safety, Cleveland, Ohio

## Abstract

**Introduction:**

Intravenous haloperidol has been shown to decrease milligram morphine equivalents (MME) of analgesia and reduce hospital admissions for diabetic gastroparesis. The objective of this study was to evaluate whether haloperidol decreases MME for the treatment of non-specific abdominal pain diagnoses in the emergency department (ED), including gastroparesis, cyclic vomiting, cannabinoid hyperemesis syndrome, and unspecified abdominal pain. The primary outcome compared the difference in MME between encounters. Secondary outcomes included admission rate, pain scores, length of stay, rescue therapy administration, and adverse effects.

**Methods:**

This retrospective chart review included patients ≥ 18 years old who presented to the ED. Patients must have had ≥ 2 ED encounters for abdominal pain, one in which they received conventional therapy with opioids (C-encounter), and the other in which they received haloperidol (H-encounter). Agitated patients were excluded. Seventy-five patients were needed to detect a 3 MME difference with 80% power and two-sided alpha of 0.05.

**Results:**

We analyzed 107 patients with self-matched encounters. The median dose of haloperidol administered was 5.0 milligrams (mg) (interquartile range [IQR] 2.0 – 5.0). C-encounters had significantly more MME administered than H-encounters (median 5.7 mg [IQR 4.0 – 8.0] vs 0.0 mg [IQR 0.0 – 2.5], P < 0.001). These results remained significant despite route of haloperidol administration. C-encounters had higher rates of rescue therapy administration than H-encounters, (56% vs 33.6%, P < 0.001). There were higher rates of ketorolac administration in the H-encounter (P = 0.02).

**Conclusion:**

Encounters in which patients received haloperidol and ketorolac for abdominal pain had a statistically significant reduction in MME administered and lower rates of rescue therapy administration than encounters in which patients were treated with opioids.

## INTRODUCTION

Pain accounts for 45.4% of all emergency department (ED) visits in the United States. Abdominal pain accounts for up to 8% of those visits.^1^,^2^ In 2016, Cervellin and colleagues reported that ED encounters for adults with acute abdominal pain had an admission rate of 17% and a readmission rate just above 6% within 30 days.^3^ Not only is abdominal pain prevalent and costly, it is becoming more difficult to treat due to limited availability of conventional therapy.^4^,^5^ Analgesics, including opioids and non-steroidal anti-inflammatory drugs (NSAID), are the mainstay of therapy for abdominal pain. Opioids may be preferred because they, unlike NSAIDs, do not have the potential to mask peritoneal inflammation.^6^ Other therapies are supportive in nature and directed toward management of concurrent nausea and vomiting.

Haloperidol, a first-generation antipsychotic, antagonizes a variety of neurotransmitters in the central nervous system.^7^ The antiemetic effect of haloperidol is due mainly to its antagonism of dopamine at the D2 receptor, histamine at the H1 receptor, and acetylcholine at the muscarinic type-1 receptor in the chemoreceptor trigger zone. This antagonism attenuates nausea and vomiting.^8^ Haloperidol also has anti-emetic effects peripherally, as it non-specifically targets D1–D5 receptors in the gut, affecting blood flow and gastric motility.^9^ Lastly, haloperidol is a structural derivative of meperidine and has been linked to analgesic effects through sigma-1 receptor antagonism.^10^,^11^

Several recent studies have examined the analgesic effect of haloperidol. In 2017, Roldan and colleagues conducted a randomized, double-blind, placebo-controlled trial comparing haloperidol 5 milligrams (mg) intravenously (IV) plus conventional therapy vs placebo plus conventional therapy for the treatment of gastroparesis. They found a significant difference in their primary outcome, mean reduction in pain scores, 5.37 vs 1.11 (*P* ≤ 0.001), and nausea scores, 2.70 vs 0.72 (*P* = 0.05), at one hour.^12^ Another study, by Ramirez and colleagues, examined the opioid-sparing effect of haloperidol. They retrospectively analyzed the effect of intramuscular (IM) haloperidol plus conventional therapy vs conventional therapy alone for the treatment of diabetic gastroparesis. This trial demonstrated that the administration of haloperidol, in addition to conventional therapy, had an opioid sparing effect, with 6.75 vs 10.75 (*P* = 0.009) morphine equivalents used, and a decreased admission rate of 10% vs 27% (*P* = 0.002) when compared to conventional therapy alone.^13^

The combined antiemetic and analgesic effects of haloperidol make it an appealing alternative for the treatment of abdominal pain with concurrent nausea and vomiting, given the limited availability of medications used as conventional treatment for abdominal pain due to drug shortages.^5^,^6^ These include but are not limited to the following: ketorolac; morphine; fentanyl; hydromorphone; diphenhydramine; ondansetron; and metoclopramide. This, in addition to decreased opioid prescribing in the setting of the opioid epidemic, was the basis for the health-system implementation of “haloperidol for analgesia” emergency services protocol. The intent of this protocol was to aid in the management of patients with gastroparesis, cannabinoid hyperemesis syndrome, cyclic vomiting, and other non-specific abdominal pain diagnoses.

This protocol provided clinical decision support and monitoring parameters for providers who sought to use haloperidol for analgesia. With the implementation of this protocol came the new medical record, “haloperidol injection 5 mg/mL (HALDOL) – ANALGESIA.” Indications for therapy were included in the order instructions and the dose of 5 milligrams intravenously (mg IV) was auto-selected, with the option of changing dose and route based on provider preference. A reference link to the full protocol was included in the medication record. In this study we sought to examine the opioid-sparing effect of haloperidol when used for abdominal pain and to determine whether the effect was dependent on route of haloperidol administration.

**Population Health Research Capsule**

What do we already know about this issue?

*Non-specific abdominal pain is a common emergency department presentation, and its treatment has been complicated by drug shortages and the opioid epidemic.*

What was the research question?

*Does haloperidol administration for the treatment of unspecified abdominal pain spare morphine equivalents?*

What was the major finding of the study?

*Haloperidol, in conjunction with ketorolac, spares approximately 6 milligram morphine equivalents.*

How does this improve population health?

*Haloperidol can reduce opioid exposure and is a safe and effective means of managing unspecified abdominal pain in a population with high healthcare utilization.*

## METHODS

This was a retrospective cross-over chart-review of an electronic health record within an integrated health system. The health system is composed of large academic medical centers, regional hospitals, and freestanding EDs, accounting for approximately 700,000 patient encounters annually. This study, number 19–122, was reviewed and exempted by the institutional review board. Included patients were 18 years of age and older admitted to an ED between July 1–December 1, 2018, and administered haloperidol 2 mg-5 mg intramuscular (IM) or IV for abdominal pain (H -encounter).

Encounters with *International Classification of Diseases*, revision 10 (ICD-10) codes associated with abdominal pain were analyzed, including, but not limited to the following: non-specific abdominal pain; peptic ulcer disease; cyclic vomiting; cannabinoid hyperemesis syndrome; and reflux disease. Abdominal pain was confirmed within the ED provider note with a positive reference in “review of systems.” Patient charts were then audited for qualifying comparison encounters in which the patient received opioids as conventional therapy (C-encounter) for abdominal pain. Encounters did not qualify as a comparator if the patient was administered antipsychotics. All comparison encounters must have been separated by a minimum of three days from other hospital encounters requiring analgesia to allow for treatment washout, and a maximum of 365 days of the haloperidol encounter to minimize variability in patient presentation between encounters. We excluded the following patients from the study: allergy or sensitivity to haloperidol; chronic use of haloperidol as a prior-to-admission medication; urgent abdominal surgery; and administration of haloperidol for acute agitation secondary to delirium, psychosis, or for sedation.

### Measurements

Baseline demographic data included age, gender, time and date of ED encounter, resulting inpatient stay if applicable, ED location, repeat ED encounter within 30 days, and death. We classified EDs according to number of annual encounters. Dose, route, and resultant pain scores were also collected. Pain was documented as a 0–10 visual analogue scale and was included if the patient had a score before and at least 15 minutes after analgesic administration. If patients had more than two qualifying comparison encounters, the most recent qualifying encounter to the haloperidol encounter was used.

The primary outcome was to analyze the difference in milligram morphine equivalent (MME) administration between the self-matched H- and C-encounters. Any opioids, NSAIDs, acetaminophen, ketamine, lidocaine, and haloperidol administered during the encounters were documented and considered to be concurrent analgesia. Secondary outcomes included disposition, adverse events, difference in pain scores, ED length of stay, repeat ED encounters within 30 days, and use of rescue medications.

The “interventions” of this review were haloperidol and opioid use for the treatment of abdominal pain. We defined rescue therapy as any analgesic or antiemetic administered 30 minutes after initial haloperidol or opioid administration. Any acetaminophen, ketorolac, or antiemetic use prior to administration of the intervention was not analyzed as rescue therapy. We selected 30 minutes to allow for initial onset of medications administered and to provide a realistic time frame for symptom reassessment in ED patients. The following antiemetics were considered rescue therapy: diphenhydramine; metoclopramide; ondansetron; promethazine; and prochlorperazine. All routes of rescue therapy administration were included for analysis. Additional agents were not included due to formulary restrictions. We calculated MMEs based on an equianalgesic dosing chart.^14^ Adverse events, including arrhythmia, mental status change from start of ED encounter, seizure, dystonic reaction, and respiratory depression were recorded per nursing documentation and the medication administration record. We defined respiratory depression as respiratory rate less than 12 breaths per minute within one hour after opioid administration.

Seventy-five patients with self-matched encounters were needed to detect a difference of three MMEs with 80% power and a two-sided alpha of 0.05.^13^ Ordinal variables were compared using the Wilcoxon signed-rank test or paired t-test and categorical variables were compared using McNemar’s test. Data was expressed as medians with interquartile ranges (IQR) if data was nonparametric, as means with confidence intervals (CI) if data was parametric, or numbers and percentages of patients, as appropriate. We performed all data analysis using open-source statistical software R Commander (developed by J. Fox), R package version 3.5–3 (R Foundation for Statistical Computing, Zurich).

## RESULTS

A total of 504 patients qualified for chart review based on diagnosis audit and haloperidol administration. Breakdown of excluded patients was as follows: 218 patients lacked a qualifying comparison encounter; 160 patients had documented agitation or altered mental status; 15 patients lacked documentation of abdominal pain in their review of systems; and four patients were admitted for urgent abdominal surgery. The remaining 107 patients were included for review. Patients were administered haloperidol for the following diagnoses: cyclic vomiting; colitis/diverticulitis; gastroparesis; pancreatitis; gastroesophageal reflux disease (GERD); and unspecified abdominal pain. The diagnoses attached to the encounters were not mutually exclusive and no diagnosis predominated significantly, although many patients had concurrent diagnoses of GERD and unspecified abdominal pain.

Seventy percent of patients were women and mean age was 41 years old. The median haloperidol dose administered in the H-encounter was 5.0 mg (IQR 2.0 – 5.0). More patients were administered haloperidol IV than IM, 81.3% vs 18.7%, respectively. Seventy-nine patients, or 73.8%, had their H-encounter chronologically after their C-encounter. Encounters at ED locations with greater than 50,000 annual visits accounted for 46% and 45% of the H-encounters and C-encounters, respectively. Encounters at locations with 20,000–50,000 annual visits accounted for 40% and 39%, respectively. Encounters at locations with less than 20,000 annual visits accounted for 14% and 16%, respectively.

H-encounters had a statistically significant reduction in MME administered when compared to C-encounters, (median 0.0 [IQR 0.0 – 2.5] vs 5.7 [IQR 4.0 – 8.0]; *P* < 0.001). This opioid-sparing effect remained significant despite route of haloperidol administration. The median MME given with IV haloperidol was 0.00 mg (IQR 0.0–4.0) vs 5.8 mg (IQR 4.0–8.0); *P* < 0.001 in the comparison C-encounter. The median MME given with IM haloperidol was 0.0 mg (IQR 0.0–0.0) vs 5.0 (IQR 3.3–8.0) in the comparison C-encounter; *P* < 0.001. H-encounters were associated with significantly lower rates of rescue therapy administration than C-encounters. This remained significant when separately analyzing rescue antiemetic and analgesic use. Six patients who received haloperidol for abdominal pain required a repeat dose. Haloperidol was not used as rescue therapy for any of the C-encounters.

Patients had significantly less opioid use in the H-encounter than in the C-encounter, 47.2% vs 100% (*P* < 0.001) but received significantly more ketorolac, 38.9% vs 14.0%; (*P* = 0.02). Mean dose of ketorolac administered in these H-encounters was 17.1 mg (CI 15.1 – 19.2). We conducted a post-hoc analysis of IM and IV ketorolac administration in H-encounters. Twenty-five percent of patients received haloperidol > 30 minutes before ketorolac, 25% of patients had concurrent administration of haloperidol and ketorolac, and 50% of patients received haloperidol > 30 minutes after the administration of ketorolac.

There were no statistically significant differences in ED length of stay, admission rate, mean pain score difference between encounters, adverse events, and 30-day repeat encounters. There were no adverse events in the H-encounters and one adverse event of mental status change in the C-encounter. Although 30-day repeat encounters related to abdominal pain were lower with patients who received haloperidol, the difference was not significant (Table 1).

## DISCUSSION

In this study, haloperidol was associated with a greater opioid-sparing effect than previous literature had demonstrated. Prior studies showed that patients who received haloperidol for abdominal pain were spared approximately 4 MME, while patients in this study were spared approximately 6 MME. Unlike what was reported in previous literature, haloperidol was not associated with a significant decrease in admission rate or pain scores when compared to conventional therapy with opioids. This is likely because previous studies did not use paired comparators, while in this study we used McNemar’s test to analyze paired, nonparametric data.

Patients who were administered haloperidol for abdominal pain needed significantly less rescue analgesia and rescue antiemetics than patients who were treated with opioids. This may be correlated to the inherent antiemetic properties of haloperidol. No patients administered concurrent lidocaine or ketamine met inclusion criteria for data analysis. Because haloperidol is not a first-line agent for acute analgesia, it is typically administered to patients who are refractory to other agents. This may explain the significant decrease in rescue therapy after haloperidol administration; many of the other agents were given prior. Although ketorolac was administered more frequently in the H-encounter, 50% of these patients received haloperidol greater than 30 minutes after the administration of ketorolac, which may suggest their pain was refractory to NSAID therapy. Another 25% of these patients had concurrent administration of ketorolac and haloperidol.

This study was multicenter, with sites varying from large academic institutions to freestanding EDs, which increases generalizability of the results. Other strengths of the study include a large patient population, a wide variety of emergent settings, and the fact that all patients were self-matched. Data was collected by a single researcher, and primary endpoints are objective and well-defined, which minimizes variability in documentation. Self-matching patients decreases variability in comorbid conditions, prior to admission medications, and perception of pain. Lastly, the time frame selected for patient presentation allowed complete treatment washout between encounters.

## LIMITATIONS

Although the time frame for qualifying comparison encounters was selected to minimize variability in presentation, diagnosis, and prior to admission medications, patients may have had differences between encounters. Several secondary outcomes relied on nursing documentation in the electronic health record, including pain score changes and adverse effects. Documentation of pain scores was sporadic. Only 21 out of 107 patients in the H-encounter had pre- and post-analgesic pain scores recorded, compared to 101 out of 107 in the C-encounter. This disparity is likely due to lack of a best practice alert prompting nursing staff to document pain scores after haloperidol administration, unlike when they administer opioids. Because adverse drug events were identified with nursing notes, their occurrence may be under-reported in this study. Repeat encounters within 30 days may have been underestimated, as only repeat encounters within the health system are visible. Higher rates of administration of ketorolac in the H-encounter may have confounded the opioid-sparing effect and need for rescue analgesia.

## CONCLUSION

This is the largest study to date analyzing haloperidol for the treatment of abdominal pain. It demonstrates that both IM and IV haloperidol, in conjunction with ketorolac, significantly reduces the amount of MMEs used for the treatment of abdominal pain and significantly decreases the need for rescue therapy when compared to conventional opioid therapy. These findings allows us to reduce opioid exposure, treat acute pain despite drug shortages, and demonstrate the safety and efficacy of managing chronic abdominal pain in a population with baseline high healthcare utilization.

## Figures and Tables

**Table 1:** Safety outcomes and concurrent analgesia.

Variable	Haloperidol encounter n = 107	Conventional encounter n = 107	P-value
Adverse events, n (%)	0 (0)	1 (0.01)	0.32
30-day repeat encounter, n (%)	47 (43.9)	60 (56.0)	0.18
Repeat encounter related to abdominal pain, n (%)	33 (70.2)	46 (76.7)	0.08
Encounter length of stay, hours (95% Cl)	5.1 (4.6–5.5)	5.3 (4.8–5.8)	0.52
Concurrent analgesia, n (%)	60 (56)	107 (100)	< 0.001
Opioids, n (%)	34 (31.7)	107 (100)	< 0.001
Ketorolac, n (%)	28 (38.9)	15 (14.0)	0.02
Acetaminophen, n (%)	6 (5.6)	3 (2.8)	0.26
Rescue therapy, n (%)	36 (33.6)	60 (56.0)	0.01
Antiemetics, n (%)	22 (20.5)	37 (34.6)	0.05
Analgesics, n (%)	22 (20.5)	48 (44.8)	< 0.001
Pain score decrease†, (95% CI)	3.1 (2.2–4.0)	3.0 (2.4–3.6)	0.93

† n = 21 for haloperidol encounter, n = 101 for conventional encounter.

*CI*, confidence interval.
